# 2-Amino-6-(2,4-dichloro­phen­yl)-4-oxo-3,5-diphenyl­cyclo­hex-2-enecarbonitrile

**DOI:** 10.1107/S1600536810035567

**Published:** 2010-09-18

**Authors:** A. Jahubar Ali, S. Athimoolam, S. Asath Bahadur, V. P. Alex Raja

**Affiliations:** a Department of Science and Humanities, National College of Engineering, Maruthakulam, Tirunelveli 627 151, India; b Department of Physics, University College of Engineering Nagercoil, Anna University of Technology Tirunelveli, Nagercoil 629 004, India; c Department of Physics, Kalasalingam University, Anand Nagar, Krishnan Koil 626 190, India; d Department of Organic Chemistry, Madurai Kamaraj University, Madurai 625 021, India

## Abstract

In the title compound, C_25_H_18_Cl_2_N_2_O, the cyclo­hexene ring has a sofa conformation. All the substituents in the cyclo­hexene ring, except the cyano group (which is axial) occupy equatorial positions. The crystal structure is stabilized through N—H⋯O hydrogen bonds, forming a chain extending along the *b* axis and through C—H⋯N and C—H⋯Cl inter­actions. It is remarkable that only one of the amino H atoms is involved in hydrogen bonding.

## Related literature

For the synthesis of the title compound, see: Rodriguez & Dulcere (1993 ▶).
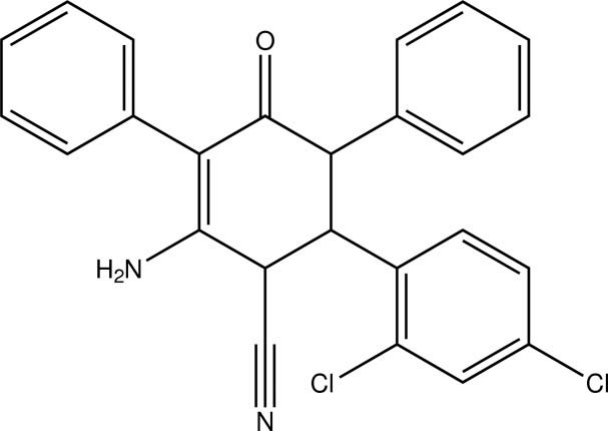


## Experimental

### 

#### Crystal data

C_25_H_18_Cl_2_N_2_O
*M* *_r_* = 433.31
Monoclinic, 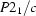

*a* = 10.8650 (9) Å
*b* = 14.0010 (3) Å
*c* = 14.3021 (6) Å
β = 94.697 (10)°
*V* = 2168.3 (2) Å^3^
*Z* = 4
Mo *K*α radiation
μ = 0.32 mm^−1^
*T* = 293 K
0.21 × 0.18 × 0.12 mm

#### Data collection

Bruker SMART APEX CCD area-detector diffractometer
18613 measured reflections
3269 independent reflections
2457 reflections with *I* > 2σ(*I*)
*R*_int_ = 0.041
θ_max_ = 23.7°

#### Refinement

*R*[*F* ^2^ > 2σ(*F* ^2^)] = 0.041
*wR*(*F* ^2^) = 0.127
*S* = 1.07
3269 reflections
271 parameters
H-atom parameters constrained
Δρ_max_ = 0.19 e Å^−3^
Δρ_min_ = −0.24 e Å^−3^

### 

Data collection: *SMART* (Bruker, 2001 ▶); cell refinement: *SAINT* (Bruker, 2001 ▶); data reduction: *SAINT*; program(s) used to solve structure: *SHELXTL/PC* (Sheldrick, 2008 ▶); program(s) used to refine structure: *SHELXTL/PC*; molecular graphics: *PLATON* (Spek, 2009 ▶); software used to prepare material for publication: *SHELXTL/PC*.

## Supplementary Material

Crystal structure: contains datablocks global, I. DOI: 10.1107/S1600536810035567/bt5346sup1.cif

Structure factors: contains datablocks I. DOI: 10.1107/S1600536810035567/bt5346Isup2.hkl

Additional supplementary materials:  crystallographic information; 3D view; checkCIF report

## Figures and Tables

**Table 1:** Hydrogen-bond geometry (Å, °)

*D*—H⋯*A*	*D*—H	H⋯*A*	*D*⋯*A*	*D*—H⋯*A*
N2—H2*B*⋯O1^i^	0.86	1.91	2.759 (3)	171
C33—H33⋯N11^ii^	0.93	2.72	3.402 (4)	131
C52—H52⋯Cl2^iii^	0.93	2.97	3.897 (3)	174
C54—H54⋯N11^iv^	0.93	2.72	3.541 (4)	147

Symmetry codes: (i) 
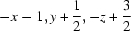
; (ii) 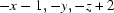; (iii) 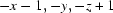; (iv) 
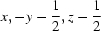
.

**Fig. 1.**
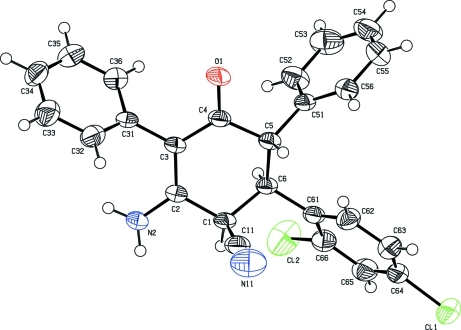
The title molecule with the atom numbering scheme. The displacement ellipsoids are shown at the 50% probability level.

**Fig. 2.**
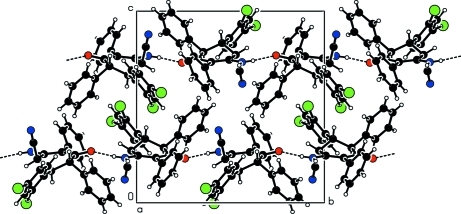
Packing diagram of the title structure viewed down the a axis. (Cl is shown in green, N in blue, O in red, C in black and H as circle)

**Fig. 3.**
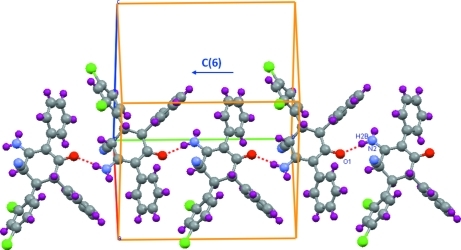
Chain C(6) motif formed through N—H···O hydrogen bonds. H-bond are drawn as dashed lines. (Cl is shown in green, N in blue, O in red, C in black and H in pink)

**Fig. 4.**
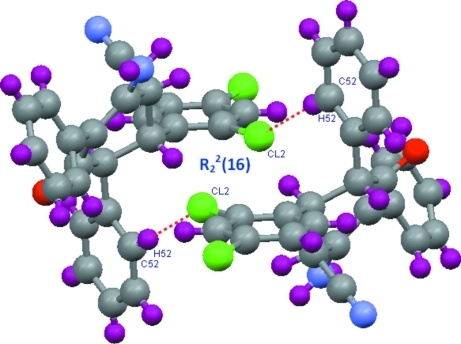
Ring R22(16) motif formed through C—H···Cl hydrogen bonds. H-bonds are drawn as dashed lines. (Cl is shown in green, N in blue, O in red, C in black and H in pink)

**Fig. 5.**
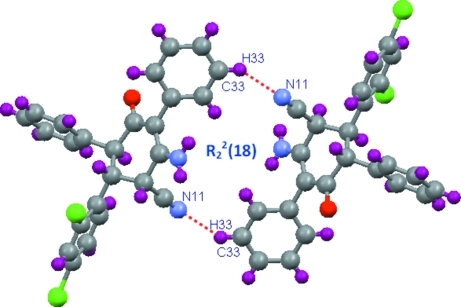
Ring R22(18) motif formed through C—H···N hydrogen bonds. H-bonds are drawn as dashed lines. (Cl is shown in green, N in blue, O in red, C in black and H in pink)

## supplementary crystallographic information

### Comment

Alkenes undergo co-halogenation reactions to afford bifunctional compounds which serve as potential synthons towards the synthesis of various heterocyclic compounds (Rodriguez & Dulcere, 1993). The regio/stereoselectivity of such addition reactions is governed by various factors, one being structural features of the alkene. We were interested in investigating some of the structure features in the title compound, which may alter the regio/stereoselectivity in addition reactions.

The molecular structure of the title compound is shown in Fig. 1. The cyclohexene ring is in a sofa conformation. Two phenyl rings are oriented with a dihedral angle of 54.8 (1)° to each other. Further, the dichlorophenyl rings are making dihedral angles of 66.7 (1)° and 84.3 (1)° with the phenyl rings of C31/C36 and C51/C56 respectively. The crystal structure is stabilized by intermolecular C—H···N, C—H···Cl and classical N—H···O hydrgeon bonds (Tab. 1). The packing diagram of the title compound is shown in Fig. 2.

A chain C(6) motif  extending along the *b* axis of the unit cell is observed through classical N—H···O hydrogen bond (Fig.3). Centrosymmetric ring *R*_2_^2^(16) motif is formed around the crystallographic inversion centre through C—H···Cl bond (Fig. 4). Further, another ring *R*_2_^2^(18) motif is observed around crystallographic inversion centre through a C—H···N bond (Fig. 5). These ring motifs are connected along *c* axis of the unit cell through another C—H···N bond [C54—H54···N11(*x*, -*y* - 1/2, *z* - 1/2)].

### Experimental

A mixture of 1,3-diphenylacetone 5 (1 mmol), 2-[(2,4-dichlorophenyl)methylene]malononitrile (1 mmol), and sodium ethoxide (2 mmol) was ground well in a mortar and pestle at ambient temperature for about 15–30 sec. Then water (50–70 ml) was added to the mixture and the product was filtered and washed with water, dried *in vacuo* and subjected to flash chromatographic purification employing flash silica gel (230–400 mesh) with petroleum ether-ethyl acetate mixture as eluent. The products were further recrystallized from ethanol-ethyl acetate mixture (1:2 *v*/*v*).

### Refinement

All the H atoms were positioned geometrically and refined using a riding model, with C—H = 0.93 Å and *U*_iso_(H) = 1.2 *U*_eq_ (parent atom).

### Crystal data

**Table:**

C_25_H_18_Cl_2_N_2_O	*F*(000) = 896
*M**_r_* = 433.31	*D*_x_ = 1.327 Mg m^−^^3^
Monoclinic, *P*2_1_/*c*	Mo *K*α radiation, λ = 0.71073 Å
Hall symbol: -P 2ybc	Cell parameters from 4312 reflections
*a* = 10.8650 (9) Å	θ = 2.1–20.1°
*b* = 14.0010 (3) Å	µ = 0.32 mm^−^^1^
*c* = 14.3021 (6) Å	*T* = 293 K
β = 94.697 (10)°	Block, colourless
*V* = 2168.3 (2) Å^3^	0.21 × 0.18 × 0.12 mm
*Z* = 4	

### Data collection

**Table:**

Bruker SMART APEX CCD area-detector diffractometer	2457 reflections with *I* > 2σ(*I*)
Radiation source: fine-focus sealed tube	*R*_int_ = 0.041
graphite	θ_max_ = 23.7°, θ_min_ = 1.9°
ω scans	*h* = −12→12
18613 measured reflections	*k* = −15→15
3269 independent reflections	*l* = −11→16

### Refinement

**Table:**

Refinement on *F*^2^	Primary atom site location: structure-invariant direct methods
Least-squares matrix: full	Secondary atom site location: difference Fourier map
*R*[*F*^2^ > 2σ(*F*^2^)] = 0.041	Hydrogen site location: inferred from neighbouring sites
*wR*(*F*^2^) = 0.127	H-atom parameters constrained
*S* = 1.07	*w* = 1/[σ^2^(*F*_o_^2^) + (0.0688*P*)^2^ + 0.6095*P*] where *P* = (*F*_o_^2^ + 2*F*_c_^2^)/3
3269 reflections	(Δ/σ)_max_ < 0.001
271 parameters	Δρ_max_ = 0.19 e Å^−^^3^
0 restraints	Δρ_min_ = −0.24 e Å^−^^3^

### Special details

**Table:**

Geometry. All e.s.d.'s (except the e.s.d. in the dihedral angle between two l.s. planes) are estimated using the full covariance matrix. The cell e.s.d.'s are taken into account individually in the estimation of e.s.d.'s in distances, angles and torsion angles; correlations between e.s.d.'s in cell parameters are only used when they are defined by crystal symmetry. An approximate (isotropic) treatment of cell e.s.d.'s is used for estimating e.s.d.'s involving l.s. planes.
Refinement. Refinement of *F*^2^ against ALL reflections. The weighted *R*-factor *wR* and goodness of fit *S* are based on *F*^2^, conventional *R*-factors *R* are based on *F*, with *F* set to zero for negative *F*^2^. The threshold expression of *F*^2^ > σ(*F*^2^) is used only for calculating *R*-factors(gt) *etc*. and is not relevant to the choice of reflections for refinement. *R*-factors based on *F*^2^ are statistically about twice as large as those based on *F*, and *R*- factors based on ALL data will be even larger.

### Fractional atomic coordinates and isotropic or equivalent isotropic displacement parameters (Å^2^)

**Table:**

	*x*	*y*	*z*	*U*_iso_*/*U*_eq_	
C1	−0.4045 (2)	0.02803 (16)	0.72770 (17)	0.0330 (6)	
H1	−0.4171	0.0873	0.6918	0.040*	
C11	−0.3249 (3)	0.04896 (18)	0.8132 (2)	0.0431 (7)	
N11	−0.2610 (3)	0.0631 (2)	0.8783 (2)	0.0732 (8)	
C2	−0.5291 (2)	−0.00720 (16)	0.75371 (18)	0.0368 (6)	
N2	−0.6097 (2)	0.06106 (15)	0.76626 (19)	0.0593 (7)	
H2A	−0.6820	0.0468	0.7825	0.071*	
H2B	−0.5901	0.1198	0.7582	0.071*	
C3	−0.5514 (2)	−0.10311 (16)	0.76488 (18)	0.0339 (6)	
C31	−0.6736 (2)	−0.13559 (16)	0.79292 (18)	0.0349 (6)	
C32	−0.7174 (3)	−0.1068 (2)	0.8762 (2)	0.0499 (7)	
H32	−0.6686	−0.0681	0.9170	0.060*	
C33	−0.8332 (3)	−0.1348 (2)	0.9000 (2)	0.0570 (8)	
H33	−0.8622	−0.1139	0.9559	0.068*	
C34	−0.9040 (3)	−0.1925 (2)	0.8420 (2)	0.0529 (8)	
H34	−0.9823	−0.2105	0.8573	0.064*	
C35	−0.8600 (3)	−0.2245 (2)	0.7603 (2)	0.0553 (8)	
H35	−0.9075	−0.2658	0.7214	0.066*	
C36	−0.7462 (2)	−0.19578 (18)	0.7358 (2)	0.0450 (7)	
H36	−0.7179	−0.2173	0.6800	0.054*	
C4	−0.4589 (2)	−0.17242 (16)	0.74985 (17)	0.0341 (6)	
O1	−0.47452 (17)	−0.25760 (11)	0.76898 (14)	0.0518 (5)	
C5	−0.3365 (2)	−0.14431 (15)	0.71259 (17)	0.0317 (6)	
H5	−0.2750	−0.1411	0.7665	0.038*	
C51	−0.2942 (2)	−0.22077 (16)	0.64729 (17)	0.0326 (6)	
C52	−0.3644 (3)	−0.2437 (2)	0.56597 (19)	0.0503 (7)	
H52	−0.4376	−0.2109	0.5501	0.060*	
C53	−0.3271 (3)	−0.3151 (2)	0.5073 (2)	0.0637 (9)	
H53	−0.3759	−0.3305	0.4529	0.076*	
C54	−0.2196 (3)	−0.3629 (2)	0.5289 (2)	0.0623 (9)	
H54	−0.1945	−0.4104	0.4891	0.075*	
C55	−0.1493 (3)	−0.3410 (2)	0.6084 (2)	0.0587 (8)	
H55	−0.0756	−0.3735	0.6233	0.070*	
C56	−0.1864 (2)	−0.27073 (18)	0.6676 (2)	0.0442 (7)	
H56	−0.1376	−0.2569	0.7224	0.053*	
C6	−0.3415 (2)	−0.04533 (15)	0.66641 (16)	0.0304 (6)	
H6	−0.3942	−0.0514	0.6078	0.037*	
C61	−0.2188 (2)	−0.00592 (16)	0.64069 (17)	0.0331 (6)	
C62	−0.1086 (2)	−0.02628 (18)	0.69160 (19)	0.0421 (7)	
H62	−0.1091	−0.0689	0.7414	0.051*	
C63	0.0019 (3)	0.01363 (19)	0.6720 (2)	0.0495 (7)	
H63	0.0744	−0.0020	0.7078	0.059*	
C64	0.0039 (2)	0.07653 (18)	0.5992 (2)	0.0447 (7)	
C65	−0.1028 (3)	0.09852 (19)	0.54492 (19)	0.0482 (7)	
H65	−0.1012	0.1407	0.4948	0.058*	
C66	−0.2121 (2)	0.05682 (18)	0.56630 (18)	0.0408 (6)	
Cl1	0.14075 (7)	0.13127 (6)	0.57487 (7)	0.0697 (3)	
Cl2	−0.34352 (8)	0.08729 (7)	0.49620 (6)	0.0767 (3)	

### Atomic displacement parameters (Å^2^)

**Table:**

	*U*^11^	*U*^22^	*U*^33^	*U*^12^	*U*^13^	*U*^23^
C1	0.0406 (14)	0.0178 (12)	0.0411 (15)	−0.0015 (10)	0.0056 (12)	0.0034 (10)
C11	0.0529 (17)	0.0276 (14)	0.0496 (19)	−0.0052 (12)	0.0085 (15)	−0.0023 (13)
N11	0.091 (2)	0.0676 (19)	0.0584 (18)	−0.0100 (16)	−0.0111 (17)	−0.0102 (15)
C2	0.0385 (14)	0.0216 (13)	0.0509 (16)	−0.0003 (11)	0.0069 (12)	−0.0025 (11)
N2	0.0484 (14)	0.0201 (11)	0.113 (2)	0.0014 (10)	0.0288 (14)	0.0001 (12)
C3	0.0390 (14)	0.0188 (12)	0.0445 (15)	−0.0007 (10)	0.0072 (11)	−0.0015 (10)
C31	0.0398 (14)	0.0173 (12)	0.0481 (16)	−0.0011 (10)	0.0057 (12)	0.0014 (11)
C32	0.0500 (17)	0.0442 (16)	0.0569 (19)	−0.0121 (13)	0.0128 (14)	−0.0087 (14)
C33	0.0554 (18)	0.059 (2)	0.059 (2)	−0.0105 (16)	0.0200 (15)	−0.0041 (16)
C34	0.0399 (15)	0.0469 (17)	0.073 (2)	−0.0075 (13)	0.0103 (15)	0.0120 (15)
C35	0.0475 (17)	0.0484 (18)	0.069 (2)	−0.0146 (14)	0.0020 (16)	−0.0070 (15)
C36	0.0460 (16)	0.0388 (15)	0.0505 (17)	−0.0032 (13)	0.0063 (13)	−0.0033 (13)
C4	0.0414 (14)	0.0208 (13)	0.0401 (15)	−0.0012 (11)	0.0043 (11)	−0.0006 (10)
O1	0.0584 (12)	0.0189 (10)	0.0814 (14)	0.0015 (8)	0.0262 (10)	0.0072 (9)
C5	0.0370 (13)	0.0220 (12)	0.0352 (14)	0.0006 (10)	−0.0015 (11)	0.0004 (10)
C51	0.0376 (14)	0.0218 (12)	0.0388 (15)	−0.0035 (10)	0.0065 (12)	0.0008 (10)
C52	0.0589 (17)	0.0416 (16)	0.0487 (18)	0.0059 (14)	−0.0059 (14)	−0.0082 (14)
C53	0.091 (3)	0.057 (2)	0.0431 (18)	−0.0098 (19)	0.0013 (17)	−0.0123 (15)
C54	0.083 (2)	0.0398 (17)	0.068 (2)	−0.0021 (17)	0.0296 (19)	−0.0153 (16)
C55	0.0547 (18)	0.0407 (17)	0.083 (2)	0.0088 (14)	0.0164 (17)	−0.0076 (16)
C56	0.0407 (15)	0.0344 (15)	0.0573 (18)	0.0007 (12)	0.0024 (13)	−0.0041 (13)
C6	0.0348 (13)	0.0208 (12)	0.0354 (14)	−0.0018 (10)	0.0008 (11)	0.0017 (10)
C61	0.0396 (14)	0.0224 (12)	0.0376 (15)	−0.0010 (10)	0.0054 (11)	−0.0021 (11)
C62	0.0411 (15)	0.0352 (14)	0.0498 (17)	−0.0036 (12)	0.0021 (13)	0.0074 (12)
C63	0.0398 (15)	0.0422 (16)	0.066 (2)	−0.0031 (13)	0.0035 (14)	−0.0018 (15)
C64	0.0464 (17)	0.0334 (15)	0.0574 (18)	−0.0070 (12)	0.0222 (14)	−0.0106 (13)
C65	0.063 (2)	0.0375 (15)	0.0464 (17)	−0.0061 (14)	0.0205 (15)	0.0027 (13)
C66	0.0467 (15)	0.0349 (14)	0.0405 (15)	−0.0003 (12)	0.0026 (12)	0.0035 (12)
Cl1	0.0587 (5)	0.0600 (5)	0.0957 (7)	−0.0188 (4)	0.0390 (4)	−0.0138 (4)
Cl2	0.0674 (6)	0.0881 (7)	0.0720 (6)	−0.0043 (5)	−0.0109 (4)	0.0438 (5)

### Geometric parameters (Å, °)

**Table:**

C1—C11	1.469 (4)	C5—H5	0.9800
C1—C2	1.516 (3)	C51—C56	1.375 (3)
C1—C6	1.546 (3)	C51—C52	1.375 (4)
C1—H1	0.9800	C52—C53	1.388 (4)
C11—N11	1.132 (3)	C52—H52	0.9300
C2—N2	1.319 (3)	C53—C54	1.358 (5)
C2—C3	1.376 (3)	C53—H53	0.9300
N2—H2A	0.8600	C54—C55	1.353 (5)
N2—H2B	0.8600	C54—H54	0.9300
C3—C4	1.426 (3)	C55—C56	1.380 (4)
C3—C31	1.489 (3)	C55—H55	0.9300
C31—C36	1.376 (3)	C56—H56	0.9300
C31—C32	1.379 (4)	C6—C61	1.516 (3)
C32—C33	1.387 (4)	C6—H6	0.9800
C32—H32	0.9300	C61—C62	1.379 (3)
C33—C34	1.353 (4)	C61—C66	1.386 (3)
C33—H33	0.9300	C62—C63	1.374 (4)
C34—C35	1.373 (4)	C62—H62	0.9300
C34—H34	0.9300	C63—C64	1.365 (4)
C35—C36	1.371 (4)	C63—H63	0.9300
C35—H35	0.9300	C64—C65	1.376 (4)
C36—H36	0.9300	C64—Cl1	1.734 (3)
C4—O1	1.238 (3)	C65—C66	1.380 (4)
C4—C5	1.524 (3)	C65—H65	0.9300
C5—C51	1.517 (3)	C66—Cl2	1.730 (3)
C5—C6	1.534 (3)		
			
C11—C1—C2	109.7 (2)	C56—C51—C52	117.7 (2)
C11—C1—C6	110.3 (2)	C56—C51—C5	121.7 (2)
C2—C1—C6	111.65 (18)	C52—C51—C5	120.7 (2)
C11—C1—H1	108.4	C51—C52—C53	120.7 (3)
C2—C1—H1	108.4	C51—C52—H52	119.7
C6—C1—H1	108.4	C53—C52—H52	119.7
N11—C11—C1	177.9 (3)	C54—C53—C52	120.4 (3)
N2—C2—C3	124.5 (2)	C54—C53—H53	119.8
N2—C2—C1	114.5 (2)	C52—C53—H53	119.8
C3—C2—C1	121.0 (2)	C55—C54—C53	119.7 (3)
C2—N2—H2A	120.0	C55—C54—H54	120.1
C2—N2—H2B	120.0	C53—C54—H54	120.1
H2A—N2—H2B	120.0	C54—C55—C56	120.2 (3)
C2—C3—C4	120.9 (2)	C54—C55—H55	119.9
C2—C3—C31	119.9 (2)	C56—C55—H55	119.9
C4—C3—C31	119.2 (2)	C51—C56—C55	121.4 (3)
C36—C31—C32	118.0 (2)	C51—C56—H56	119.3
C36—C31—C3	120.5 (2)	C55—C56—H56	119.3
C32—C31—C3	121.5 (2)	C61—C6—C5	115.67 (19)
C31—C32—C33	121.0 (3)	C61—C6—C1	109.56 (18)
C31—C32—H32	119.5	C5—C6—C1	110.94 (18)
C33—C32—H32	119.5	C61—C6—H6	106.7
C34—C33—C32	120.0 (3)	C5—C6—H6	106.7
C34—C33—H33	120.0	C1—C6—H6	106.7
C32—C33—H33	120.0	C62—C61—C66	116.0 (2)
C33—C34—C35	119.8 (3)	C62—C61—C6	122.6 (2)
C33—C34—H34	120.1	C66—C61—C6	121.3 (2)
C35—C34—H34	120.1	C63—C62—C61	122.9 (3)
C36—C35—C34	120.4 (3)	C63—C62—H62	118.5
C36—C35—H35	119.8	C61—C62—H62	118.5
C34—C35—H35	119.8	C64—C63—C62	119.1 (3)
C35—C36—C31	120.9 (3)	C64—C63—H63	120.4
C35—C36—H36	119.6	C62—C63—H63	120.4
C31—C36—H36	119.6	C63—C64—C65	120.6 (2)
O1—C4—C3	120.7 (2)	C63—C64—Cl1	120.4 (2)
O1—C4—C5	117.7 (2)	C65—C64—Cl1	119.0 (2)
C3—C4—C5	121.5 (2)	C64—C65—C66	118.7 (3)
C51—C5—C4	110.51 (19)	C64—C65—H65	120.6
C51—C5—C6	111.97 (19)	C66—C65—H65	120.6
C4—C5—C6	112.53 (19)	C65—C66—C61	122.6 (2)
C51—C5—H5	107.2	C65—C66—Cl2	116.9 (2)
C4—C5—H5	107.2	C61—C66—Cl2	120.5 (2)
C6—C5—H5	107.2		
			
C2—C1—C11—N11	121 (8)	C56—C51—C52—C53	0.3 (4)
C6—C1—C11—N11	−2(8)	C5—C51—C52—C53	−178.5 (2)
C11—C1—C2—N2	87.0 (3)	C51—C52—C53—C54	−0.8 (5)
C6—C1—C2—N2	−150.4 (2)	C52—C53—C54—C55	0.5 (5)
C11—C1—C2—C3	−91.5 (3)	C53—C54—C55—C56	0.1 (5)
C6—C1—C2—C3	31.1 (3)	C52—C51—C56—C55	0.4 (4)
N2—C2—C3—C4	180.0 (3)	C5—C51—C56—C55	179.2 (2)
C1—C2—C3—C4	−1.7 (4)	C54—C55—C56—C51	−0.6 (4)
N2—C2—C3—C31	0.0 (4)	C51—C5—C6—C61	−63.6 (3)
C1—C2—C3—C31	178.4 (2)	C4—C5—C6—C61	171.2 (2)
C2—C3—C31—C36	120.0 (3)	C51—C5—C6—C1	170.83 (19)
C4—C3—C31—C36	−59.9 (3)	C4—C5—C6—C1	45.6 (3)
C2—C3—C31—C32	−60.1 (4)	C11—C1—C6—C61	−58.9 (2)
C4—C3—C31—C32	120.0 (3)	C2—C1—C6—C61	178.81 (19)
C36—C31—C32—C33	−2.4 (4)	C11—C1—C6—C5	70.0 (2)
C3—C31—C32—C33	177.7 (3)	C2—C1—C6—C5	−52.3 (3)
C31—C32—C33—C34	1.2 (5)	C5—C6—C61—C62	−31.7 (3)
C32—C33—C34—C35	1.1 (5)	C1—C6—C61—C62	94.5 (3)
C33—C34—C35—C36	−2.1 (5)	C5—C6—C61—C66	151.3 (2)
C34—C35—C36—C31	0.8 (4)	C1—C6—C61—C66	−82.4 (3)
C32—C31—C36—C35	1.4 (4)	C66—C61—C62—C63	1.0 (4)
C3—C31—C36—C35	−178.7 (2)	C6—C61—C62—C63	−176.1 (2)
C2—C3—C4—O1	172.7 (2)	C61—C62—C63—C64	0.2 (4)
C31—C3—C4—O1	−7.4 (4)	C62—C63—C64—C65	−1.3 (4)
C2—C3—C4—C5	−5.6 (4)	C62—C63—C64—Cl1	177.9 (2)
C31—C3—C4—C5	174.4 (2)	C63—C64—C65—C66	1.0 (4)
O1—C4—C5—C51	38.1 (3)	Cl1—C64—C65—C66	−178.2 (2)
C3—C4—C5—C51	−143.6 (2)	C64—C65—C66—C61	0.4 (4)
O1—C4—C5—C6	164.1 (2)	C64—C65—C66—Cl2	179.7 (2)
C3—C4—C5—C6	−17.7 (3)	C62—C61—C66—C65	−1.4 (4)
C4—C5—C51—C56	−117.1 (3)	C6—C61—C66—C65	175.8 (2)
C6—C5—C51—C56	116.6 (2)	C62—C61—C66—Cl2	179.4 (2)
C4—C5—C51—C52	61.7 (3)	C6—C61—C66—Cl2	−3.5 (3)
C6—C5—C51—C52	−64.6 (3)		

### Hydrogen-bond geometry (Å, °)

**Table:**

*D*—H···*A*	*D*—H	H···*A*	*D*···*A*	*D*—H···*A*
N2—H2B···O1^i^	0.86	1.91	2.759 (3)	171
C33—H33···N11^ii^	0.93	2.72	3.402 (4)	131
C52—H52···Cl2^iii^	0.93	2.97	3.897 (3)	174
C54—H54···N11^iv^	0.93	2.72	3.541 (4)	147

Symmetry codes: (i) −*x*−1, *y*+1/2, −*z*+3/2; (ii) −*x*−1, −*y*, −*z*+2; (iii) −*x*−1, −*y*, −*z*+1; (iv) *x*, −*y*−1/2, *z*−1/2.
